# Medicine

**Published:** 1907-06

**Authors:** George Parker


### Progress of the Medical Sciences.

### MEDICINE.

Pleural effusions present so many difficulties in diagnosis, that every additional symptom is welcome which really aids in revealing their presence. In children especially the physical signs are often hopeless, and we may be compelled to make exploratory punctures, which are themselves by no means reliable. Much discussion has taken place over the **paravertebral triangle of dulness on the opposite side** to an effusion which was first described by Grocco in 1902. Thayer and Fabyan[2] found this dulness crossing the median line and extending over to the healthy side in thirty cases out of thirty-two, one of the other cases being an interlobar empyæma, where it could hardly be expected to be present. Over the vertebræ themselves the dulness is marked to about the level of the flatness caused by the effusion on the affected side. The base of the triangle extends beyond them for two to seven centimetres, and a line joining the end of the base to the highest dull vertebral hypophysis forms the third side. In this area the respiratory sounds may be suppressed, and those sounds which are heard are tubular or nasal in character. It is usually larger in right-sided effusions, and is particularly valuable in encapsulated ones where the diagnosis is difficult. In pneumonia a similar well-marked area of dulness rarely occurs, and if present it does not alter or vanish when the patient lies on

[2] *Am. J. M. Sc.*, 1907, cxxxiii. 14.

the affected side. In effusion, on the contrary, this dulness at once changes with the posture. The writers think that the fluid lies against and in front of the vertebræ and dulls the vibrations which would be conveyed through them. Pieraccini noted a similar but hyper-resonant area on the healthy side in a case of pneumo-thorax. I have endeavoured to subject this last observation to a test, but though the resonance over the vertebræ was marked, it was difficult to satisfy oneself that there was real hyper-resonance there, or over the healthy side in the area under discussion.

**Hypertension.**—Some time ago reference was made in these columns to the terrible mortality due to the common forms of apoplexy, that is chiefly to cerebral hemorrhage and thrombosis. Since that time considerable progress has been made both in the way of prevention and treatment. The estimation of blood pressure by exact instruments has become more common, and much research has been devoted to the means of preventing or reducing excessive tension. Since many cases of this over-tension have been found unconnected with nephritis or any known disease, efforts have been made to map out the course and tendencies of this "idiopathic" form, with the result that a fairly well-marked group of symptoms has been recognised, though the underlying cause is still uncertain. A theory has been put forward by Loeb[1] that hypertension is an effort towards self-preservation, though at a serious cost. The blood supply of certain parts of the organism is an absolute necessity, and to maintain this the pressure is automatically raised, even though other structures are imperilled. Cushing, indeed, propounded this explanation of the extraordinary blood tension found in compression of the brain, viewing it as an effort to maintain the blood supply of the medulla. In kidney disease and other instances the rise of pressure has been usually regarded as the result of the irritation of various toxins on vaso-motor nerves, or on the muscular wall of the arterioles. Increased suprarenal secretion, lessened thyroid activity, or excessive viscosity of the blood, have also been invoked as causes. Now Loeb finds that in kidney disease hypertension appears chiefly in those forms where the glomeruli are most affected, and he regards it as a means of maintaining their blood supply, and hence the functional activity of the kidneys, by a reflex which raises the general blood supply through the cerebro-spinal centres. Similar reflexes are invoked in other cases of hypertension. Certainly the supply of blood to each vital organ is all essential. In orthopnœa, as Leonard Williams says, the patient sits up to facilitate the efflux of venous blood from the medulla. Still, there seems little direct proof of these reflexes, and there is much to be said on the other side from the fact that certain substances in the blood stream can without

[1] *Deutsches Arch. f. klin. Med.*, 1905, lxxxv. 348.

doubt act directly on the muscular coat. Arterio-sclerosis is more often a result than a cause of high tension, as most observers now agree, and sometimes neither this nor any other condition can be detected as a cause. Though the patient be abstemious and placid, with healthy kidneys and vessels, the blood pressure may reach 200 mm. He may suffer from dyspnœa on slight efforts, attacks of vertigo, somnolence and palpitation.[1] The pulse rate shows no difference when he is standing and lying down, the aortic second sound is accentuated and the apex displaced outwards. Sooner or later the heart, labouring under its load, breaks down with a leaking valve, perhaps the aorta dilates, or a cerebral hemorrhage takes place, and the patient is killed or crippled for life. Clearly it is important to prevent this conclusion, either by discovering the cause of the evil, or, if this is impossible, by warding off the secondary results. Benefit is often obtained by reducing the quantity of the food, and especially the nitrogenous elements of it, and by prohibiting tea, coffee and tobacco. Oliver, at the meeting of the Therapeutical Society, advocated in some cases a lacto-vegetarian diet with the exclusion of salt. The drinking water should contain a minimum of calcium salts. Among other vaso-depressants certain benzene derivatives appeared to be useful. Senator[2] appears to rely upon similar dietetic treatment, and thinks that iodine preparations are chiefly valuable from their lessening the viscosity of the blood. He prefers to give them in combination with nitrites. Thus he administers potassium iodide with sodium nitrite for considerable periods. Others employ nitrites only on special emergencies, and trust chiefly to careful diet with the frequent administration of salines and blue pill.

Huchard[3] claims that aneurysms are best treated by thus lowering the blood pressure, and reports some instances of success. He employs a strict diet with a minimum of meat and extractives, tea, coffee, stimulants, and tobacco, and enforces rest. Too much value has, he thinks, been given to the iodides, and he would rely more upon the use of nitrites. From time to time he gives a course of milk diet with theobromine to eliminate vaso-constrictor toxins.

One of the most important points in connection with hypertension is the treatment which should be adopted when the heart gives way under the strain of high blood pressure. Leonard Williams, for instance,[4] insists that to give heart tonics in mitral regurgitation due to obstruction in the peripheral vessels is merely to whip a tired horse, but that if we lower the tension the heart will recover its tone. Evacuants, theobromine and rest in bed, and vaso-dilators would then be our chief remedies. On the other hand, some writers, such as T. C. Janeway,[5] do not hesitate to

[1] Leonard Williams, *Clin. J.*, 1907, xxx. 29. [2] *Folia Therap.*, 1907, i. 40.

[3] *J. d. Praticiens*, 1906, xx. 307. [4] *Loc. cit.*, p. 39.

[5] *Am. J. M. Sc.*, 1907, cxxxiii. 54.

employ digitalis in the failing heart due to hypertension, though in a sudden failure of the left ventricle Janeway advises the simultaneous use of vaso-dilators and cardiac stimulants.

**The diagnosis of typhoid.**—Among other aids to diagnosis the following have been recently under discussion. The continuous loss of the abdominal reflex in the infra-umbilical region has been shown by J. D. Rolleston[1] to have considerable value if daily examination is made for it. Out of forty-five cases which he tested, he found it absent or diminished in forty-two, the three others not being examined till late in the disease. The reflex is practically always present in young people both in health and during all other diseases, except in certain affections of the nervous system and abdominal states, such as appendicitis and peritonitis. Thus Müller and Seidelmann found it in 2,999 persons out of 3,000 who were not suffering from those two groups of diseases. After the age of 50 it tends to disappear even in perfect health, and therefore it is of no use as a test for typhoid after that age. In young people, however, the reaction obtained by stroking the abdomen by a pencil disappears, or is much diminished during typhoid for a variable period averaging a week. It is not clear how early it is lost. At a very early stage it may be quite brisk, and then fades away, to reappear towards the stage of lysis. If it fails to return when the temperature is falling, a relapse may be expected, and it is therefore of some prognostic value. A persistently high temperature after the return of the reaction is due to some other cause than the typhoid itself.

A yellow pigmentation of the palms of the hands and the soles of the feet is in my own experience very frequent in typhoid, and of some diagnostic value. Though it may depend to some extent on the occupation and personal habits of the patient, we rarely see in other diseases the vivid colouring which is present in typhoid. Regis[2] attributes its recognition to Philipowicz, who found that it was peculiar to typhoid, and ascribed it to an atrophic condition of the skin due to the failure of the capillary circulation. The cause is however uncertain, but Grocco thinks it is almost always present in typhoid, especially in children and women, though less certain in men. It usually appears in the first week, disappears with convalescence, but returns during a relapse, and seems to bear no relation to the severity of the case. Bernard describes another symptom to which he attaches some value in childhood. If we palpate the ileo-cocal region with care, two or three swellings will be felt varying in size from a filbert to an almond. They are parallel to the long axis of the colon, and are perceptible about the end of the first week. It has been said that these swellings are hypertrophied Peyer's patches, and they certainly seem to correspond to the situation in which the patches are found.

[1] *Brain*, 1906, xxix. 99. [2] *M. Press and Circ.*, 1906, cxxxiii. 6.

The presence of typhoid bacilli seems almost universal in the secretions and fluids of the body. Thus they are found not only in the stools, the urine and the roseolous spots, but also in the blood, the sputa, and sometimes in the bile. Von Jaksch found them in over 94 per cent. of the splenic punctures he made, and claims that this method is free from danger and is the best means of an early diagnosis. Preble[1] notices that more reliable results are now given by blood cultures, as the technique is improved. He quotes Duffy as getting positive results in all his cases where the temperature was over 102°, but less frequently when it was lower. The difference was apparently due to the stage in the disease, and the suggestion is made that the bacilli are always present in the second and third week. Coleman and Buxton find that in 604 reported cases bacilli were met with in 453, or 75 per cent. Hirsh too concludes that bacilli are always to be found at some stage, but that they disappear about the end of third week; while Pöppelmann claims to have obtained excellent results by simply making blood smears in the ordinary manner, and staining them by the May-Grünewald method. If this is confirmed in practice, a most valuable aid to diagnosis will be gained.

George Parker.

